# RUC-Net: A Residual-Unet-Based Convolutional Neural Network for Pixel-Level Pavement Crack Segmentation

**DOI:** 10.3390/s23010053

**Published:** 2022-12-21

**Authors:** Gui Yu, Juming Dong, Yihang Wang, Xinglin Zhou

**Affiliations:** 1 Key Laboratory of Metallurgical Equipment and Control Technology, Ministry of Education, Wuhan University of Science and Technology, Wuhan 430081, China; 2 School of Mechanical and Electrical Engineering, Huanggang Normal University, Huanggang 438000, China; 3 Hubei Key Laboratory of Mechanical Transmission and Manufacturing Engineering, Wuhan University of Science and Technology, Wuhan 430081, China; 4 School of Machinery and Automation, Wuhan University of Science and Technology, Wuhan 430081, China

**Keywords:** pavement crack segmentation, convolutional neural network, U-net, scSE attention mechanism module

## Abstract

Automatic crack detection is always a challenging task due to the inherent complex backgrounds, uneven illumination, irregular patterns, and various types of noise interference. In this paper, we proposed a U-shaped encoder–decoder semantic segmentation network combining Unet and Resnet for pixel-level pavement crack image segmentation, which is called RUC-Net. We introduced the spatial-channel squeeze and excitation (scSE) attention module to improve the detection effect and used the focal loss function to deal with the class imbalance problem in the pavement crack segmentation task. We evaluated our methods using three public datasets, CFD, Crack500, and DeepCrack, and all achieved superior results to those of FCN, Unet, and SegNet. In addition, taking the CFD dataset as an example, we performed ablation studies and compared the differences of various scSE modules and their combinations in improving the performance of crack detection.

## 1. Introduction

Crack is one of the most common road surface diseases that pose a potential threat to highway safety. Regular crack detection plays a vital role in the maintenance and operation of existing buildings and infrastructure. Compared with the traditional manual visual inspection method, which is tedious, subjective, and time-consuming and exposes inspectors to dangerous working conditions [1], the automatic crack detection method based on computer vision has been widely considered by academic and industrial circles for its advantages of being safer, cheaper, more efficient, and more objective.

Automatic crack detection is always a challenging task due to the influence of stains, shadows, complex texture, uneven illumination, blurring, and multiple scenes [2]. In the past decades, scholars have proposed a variety of image-based algorithms to automatically detect cracks on concrete surfaces and pavement. In the early studies, most of the methods are based on the combination or improvement of traditional digital image processing techniques (IPTs) [3], such as thresholding [4,5,6] and edge detection [7,8,9,10]. However, these methods are generally based on the significant assumption that the intensities of crack pixels are darker than the background and usually continuous, which makes these methods difficult to use effectively in the environment of complex background noise [11,12]. In order to improve the accuracy and integrity of crack detection, the methods based on wavelet transform [13,14] are proposed to lift the crack regions. However, due to the anisotropic characteristics of wavelets, they may not deal well with cracks with large curvatures or poor continuities [2].

In recent studies, several minimal path methods [15,16] have also been used for crack detection. Although these methods make use of crack features in a global view [3] and achieve good performance, their main limitation is that seed points for path tracking need to be set in advance [17], and the calculation cost is too high for practical application.

To improve the adaptability of IPTS-based methods in the real environment, methods based on machine learning (ML) have been used for damage detection by researchers, including artificial neural network (ANN) [18,19], support vector machine (SVM) [20,21,22], random structure forest [23], AdaBoost [24], and so on. These methods have good performance but heavily rely on manual feature extraction.

More recently, the supervised deep learning methods, such as convolutional neural networks (CNNs), have achieved state-of-the-art performance in many advanced computer vision tasks, such as image recognition [25], object detection [26,27], and semantic segmentation [28,29,30]. The main advantage of deep learning is that it does not rely on expert-driven heuristic thresholds or hand-designed features and has high accuracy and robustness to image variations [31].

Unet [32], as a typical representative of semantic segmentation algorithm, has achieved great success in medical image segmentation. There are many similarities between pavement crack detection and medical image segmentation, so it is natural to apply Unet to pavement crack segmentation.

The spatial-channel squeeze and excitation (scSE) [33] attention mechanism can enhance important information features while suppressing unimportant information features in space and channels [34], which is helpful for improving the semantic segmentation effect.

Inspired by Unet and scSE, this paper proposed a U-shaped encoder–decoder semantic segmentation network for pavement crack detection combining Unet with ResNet and used the scSE attention module to enhance the crack detection effect.

The main contributions of this paper can be summarized as follows:

1. We modified Unet and proposed a residual U-shaped encoder–decoder semantic segmentation network that combined Unet with ResNet18, named RUC-Net, which achieved better detection effects than the original Unet and the other classical segmentation algorithms, such as FCN [29] and SegNet [30].
2. We integrated the scSE attention mechanism in RUC-Net. This attention module correlated the global information of cracks, effectively improving the detection effect. In addition, we experimentally compared and investigated the difference of detection performance improvement by using various scSE attention module combinations in the encoder part (downsampling stage) and the decoder part (upsampling stage).
3. We introduced the focal loss function, which could reduce the weight of easy-to-classify samples, to deal with the problem of class imbalance in crack segmentation.

The rest of the paper is organized as follows: Section 2 reviews the previous work on pavement crack detection based on deep learning. Then, in Section 3, we describe the network architecture of our model, loss function, and optimization method. Next, in Section 4, we perform experimental vitrification and discuss our method. In addition, we provide ablation studies on the scSE module and the focal loss parameter choice in Section 5. Finally, in Section 6, we summarize our work and point out its limitations.

## 2. Related Work

### 2.1. Convolutional Neural Network-Based Method

With the tremendous success of deep learning methods in various computer vision tasks, many deep convolutional neural network-based methods have been proposed for road crack detection. According to the way the crack detection problem is handled, these methods can be roughly divided into three categories, namely, pure image classification methods, object detection-based methods, and pixel-level segmentation methods [35].

#### 2.1.1. Classification

Some researchers have carried out image-level classification studies, which mainly solve the problem of determining whether a road image contains cracks and, if so, what type of cracks. Ma et al. [36] developed a deep learning method for road detection and evaluation based on convolutional neural network, Fisher vector coding, and UnderBagging random forest. Notably, they developed a way to create large-scale datasets of road images, matching Google Street View maps with government inspectors’ ratings of road surfaces on specific sections. However, this method can only determine whether the condition of a road image is good, fair, or poor. Gopalakrishnan et al. proposed to use a pretrained deep convolutional neural network model with transfer learning to automatically detect pavement cracks [37]. Xu et al. proposed an end-to-end crack detection model based on a convolutional neural network (CNN) with atrous convolution, the Atrous Spatial Pyramid Pool (ASPP) module, and depthwise separable convolution [38]. Although these methods achieved good accuracy, none of the above methods provided localization information of cracks.

The patchwise detection method, which divides the original pavement images into many small patches, is adopted by more researchers due to its two advantages. First, more data can be generated, and second, the localization information of cracks can be obtained. Zhang et al. [39] proposed a six-layer CNN network with four convolutional layers and two fully connected layers and used their convolutional neural network to train 99 × 99 × 3 small patches, which were split from 3264 × 2248 road images collected by low-cost smartphones. The output of the network was the probability of whether a small patch was a crack or not. Their study shows that deep CNNs are superior to traditional machine learning techniques, such as SVM and boosting methods, in detecting pavement cracks. Pauly et al. [40] used a self-designed CNN model to study the relationship between network depth and network accuracy and proved the effectiveness of using a deeper network to improve detection accuracy in pavement crack detection based on computer vision. In contrast with [39], which used the same number of convolution kernels in all convolution layers, Nguyen et al. [41] used a convolution neural network with an increased number of convolution kernels in each layer because the features were more generic in the early layers and more original dataset specific in later layers [42]. Eisenbach et al. [43] presented the GAPs dataset, constructed a CNN network with eight convolution layers and three full connection layers, and analyzed the effectiveness of the state-of-the-art regularization techniques. However, its network input size was 64 × 64 pixels, which was too small to provide enough context information. The same problem also existed in [44,45,46].

Cha et al. [44] trained an eight-layer CNN and used sliding window technology to detect concrete cracks. While the sliding window technology was helpful in locating the crack, it was difficult to find the best size of the sliding window because the test images may have had different sizes and scales.

#### 2.1.2. Object Detection

Although patch-level classification can generate location information, the results are rough. In order to further improve the accuracy of crack detection, the method based on object detection has attracted the attention of researchers. Object detection is to locate the object with the bounding box in the image and determine the category of the object. Nie et al. [45] put forward a crack detection model based on Faster R-CNN and adopted a transfer learning method with parameter fine-tuning to realize the detection of pavement diseases such as cracks, looseness, and deformation. Cha et al. [46] adopted the modified ZF-net as the CNN feature extractor of Faster R-CNN, which accelerated feature extraction and was more suitable for real-time detection. Maeda et al. [47] developed a road disease object detection dataset, which contained eight types of road diseases and was created by collecting a large number of road images using a low-cost vehicle-mounted smartphone. They used the advanced SSD with InceptionV2 and SSD with MobileNet to train and test the model, which provided a new low-cost way for road disease detection. In addition, Mandal et al. [48] used Yolo V2, and Hu et al. [49] used Yolo V5 for road crack detection. Similar to patch-level classification, object detection can generate crack localization information, but the important features of the cracks cannot be estimated from the generated bounding boxes [50].

#### 2.1.3. Pixel-Level Segmentation

Crack detection methods based on patch-level classification or object detection can provide fast and accurate locating and counting of the surface cracks along the specific monitored pavement section, but they are difficult to use to obtain accurate information about the length, width, severity, and other parameters of individual cracks, which are important for comprehensive pavement condition evaluation [51]. Pixel-level pavement crack detection can provide accurate crack parameters for pavement condition evaluation, so it has become the current trend of crack detection based on deep learning.

Zhang et al. put forward CrackNet [52], which is an earlier study on pixel-level crack detection based on CNN. The prominent feature of CrackNet is using a CNN model without a pooling layer to retain the spatial resolution. Fei et al. have upgraded it to Cracknet-V [53]. While CrackNet and its series versions perform well, they are primarily used for 3D road crack images, and their performances on two-dimensional (2D) road crack images have not been validated. Fan et al. [3] proposed a pixel-level structured prediction method using CNN with full connections (FC) layers, but it has the disadvantage that it requires a long inference time for testing.

In recent years, semantic segmentation using fully convolutional network and encoder–decoder has become a research focus of pixel-level segmentation, among which the pioneer methods are FCN, SegNet, and Unet.

Huang et al. [54] proposed a semantic segmentation method using fully convolutional networks (FCN) for detecting cracks and leaks in subway shield tunnels. Yang et al. [12] similarly used FCN for pixel-level detection of cracks and proposed a method for skeletonizing cracks to measure morphological features of cracks, such as crack length and width. In addition, based on FCN, a deeper network was used by Li et al. [55]. They constructed an FCN architecture by fine-tuning densenet-121 for detecting four types of surface damage: cracks, spalling, efflorescence, and holes. Unet has achieved remarkable success in the semantic segmentation of medical images, and there are similarities between crack detection and medical image segmentation, so it is natural to use Unet for crack detection. Cheng et al. [56] were some of the first to use Unet to process crack images as a whole and directly generate crack segmentation results. Jenkins et al. [57] combined Unet with patch-level methods. Lau et al. [58] proposed a Unet structure with pretrained ResNET-34 as an encoder. Bang et al. [59] proposed a pixel-level pavement crack detection network with an encoder–decoder architecture for detecting black box images. Their encoder used a residual network, and the decoder combined the skip connection method of FCN and the deconvolution techniques of SegNet and ZFnet. However, the method did not work well for detecting very fine cracks.

Similarly, based on SegNet, Zou et al. [17] proposed an end-to-end deep convolutional neural network, named DeepCrack, to fuse multi-scale deep convolutional features learned in the hierarchical convolution stage to achieve better detection results.

Yang et al. [60] proposed a feature pyramid hierarchical and hierarchical boosting network for pavement crack detection, where semantic information from deeper layers was introduced into shallow layers in a pyramidal manner for integration to enrich the features in shallow layers, thus improving detection performance.

### 2.2. Transformer-Based Method

In recent years, transformers [61,62] have made great breakthroughs in CV, and it was quickly introduced into the field of crack segmentation. Ju et al. [63] proposed TransMF, which is a transformer-based multi-scale fusion model for crack detection. The Encoder Module uses a hybrid of convolution blocks and a Swin Transformer block to model the long-range dependencies of different parts in a crack image from local and global perspectives. Qu et al. [64] proposed CrackT-net, which was a method for pavement crack segmentation that combined a CNN with the transformer. The Swin Transformer Module was used as the last feature extraction layer to obtain better global information. Wang et al. [65] put forward SegCrack, which adopted a hierarchical Transformer as the encoder and employed a top-down pathway with lateral connections as the decoder. Liu et al. [66] proposed a crack transformer encoder–decoder structure, named CrackFormer, which proposed a self-attention block and scaling-attention block for fine-grained crack detection. These transformer-based methods used the cascaded self-attention module to capture feature dependencies over long distances, so as to obtain better global information.

## 3. Proposed Method

Unet was originally designed for biomedical image segmentation, such as cell image segmentation and retinal image capillary segmentation. Although these biomedical image training datasets are generally small, Unet still achieves good segmentation results. Due to the high cost of data acquisition and marking, the dataset of crack segmentation images is usually small too. However, there are some similarities between the topological structures of crack images and biomedical images. In view of the above two points, the segmentation tasks of crack images and biomedical images have strong similarities. Therefore, the authors preferred the Unet-based network for crack image segmentation.

To further improve the segmentation performance of Unet, we first considered introducing residual modules in downsampling, which increased gradient propagation and helped to improve the generalization ability of the network. Second, we introduced the scSE attention mechanism, which could enhance important information features while suppressing unimportant information features in space and channels, so as to improve the semantic segmentation effect.

### 3.1. Network Architecture

**Figure 1:**
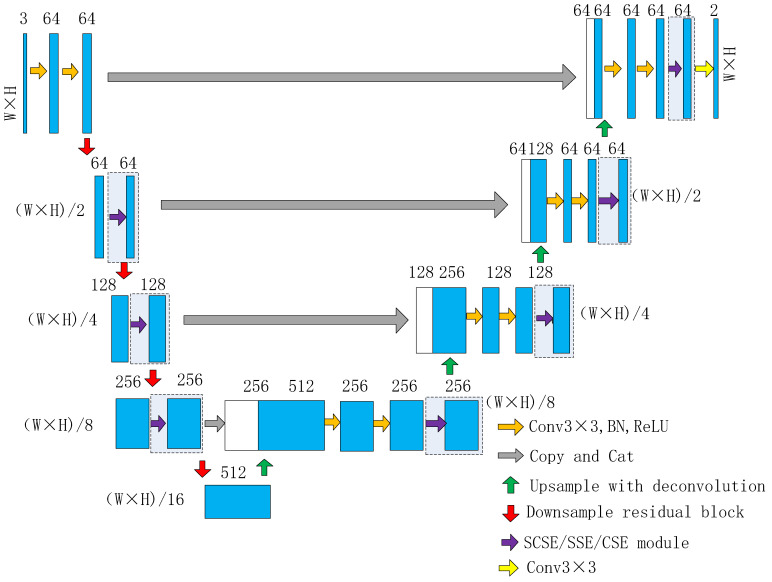
Proposed network architecture. The blue blocks and white blocks represent the feature map. The number above each block represents the number of feature channels it has. The orange arrows represent 3 × 3 convolution, BN, and ReLU layer. The red arrows represent the residual block for downsampling. The green arrows represent upsampling operation. The purple arrows represent various scSE modules. The dashed box indicates that this part can be selected as required.

**Figure 2:**
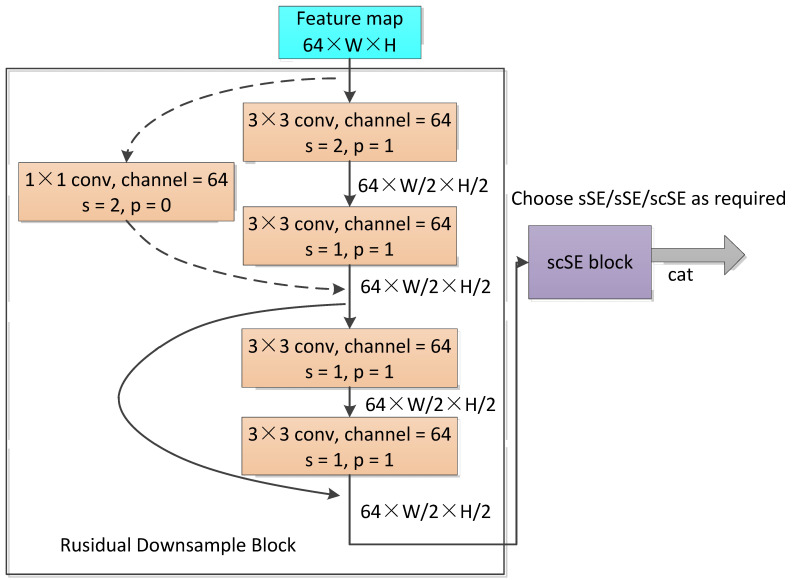
The details of the first residual downsample block and its subsequent links. The other three residual blocks are similar, except that the number of channels and the size of the feature map are different.

The network we proposed was a residual U-shaped encoder–decoder semantic segmentation network, as shown in Figure 1, called the Residual Unet Crack Network (RUC-Net). The encoder part of RUC-Net was a contraction path to capture contextual semantic information, which was modified from the encoder part of original Unet combined with Resnet18. For the encoder, we mainly modified the following:

1. The 7 × 7 convolution layer and the max pool layer at the front part of Resnet18 were removed, and the two 3 × 3 convolution layers at the front part of Unet were retained to change the number of channels from three to 64.
2. In the original Unet, after four downsamplings, the number of channels became 1024. In order to reduce the model parameters and computational complexity, unlike the original Unet, the final channel number of RUC-Net was 512 after four downsamplings. Therefore, the number of channels in the proposed network remained 64 after the first downsampling.
3. The 2 × 2 max pooling layer, which was used for downsampling, and two 3 × 3 convolution layers of the original Unet network were replaced by the residual block, which is inspired by Resnet. As shown in Figure 2, each residual block contained two basic blocks. Each basic block contained two 3 × 3 convolutions and corresponding skip connections. In the first basic block, a 3 × 3 convolution with a stride of two was used for downsampling. A total of four residual blocks were used, and the last three residual blocks were equivalent to con3_x, con4_x, and con5_x in ResNet18. The first residual block, however, used 3 × 3 convolution with a stride of two for downsampling, which was different from conv2_x of the original ResNet18, which had no downsampling. After four times of downsampling, the resolution of the feature image changed to 1/16 of the original image.

The decoder part of RUC-Net was an extended path, which upsampled the feature map and improved the resolution of the feature map step by step. The feature map obtained by each upsampling was skip connected with the feature map in the corresponding downsampling path. This skip-connection technology reused the image details that may have been lost in the encoding layers and took into account both the global information and localization accuracy of the image, so that the decoding layers could reconstruct image details more effectively [57].

### 3.2. scSE Module

Roy et al. [33] proposed an scSE module, which had three variants: sSE (‘squeezes’ along the channels and ‘excites’ spatially), cSE (‘squeezes’ along the spatial domain and ‘excites’ along the channels), and scSE (concurrent sSE and cSE). Details of their structure can be found in the original article, and their principles are briefly described below.

- The sSE module. The original feature map was changed from [C, H, W] to [1, H, W] via a 1 × 1 convolution, then activated by a sigmoid to obtain the spatial attention map, which was applied to the original feature map to recalibrate the spatial information.
- The cSE module. The feature map was first changed from [C, H, W] to [C, 1, 1] by global average pooling, then converted to a C-dimension vector after twice performing 1 × 1 convolution operations. This vector was normalized by a sigmoid and was channelwise multiplied with the original feature map to obtain a feature map recalibrated by channel information.
- The scSE module. The scSE was the combination of the sSE and cSE modules, which was essentially the parallel connection of the two modules. Specifically, after the feature map was operated through the sSE and cSE modules, we added up the two outputs to recalibrate the feature map both spatially and channelwise.

In this paper, we discuss the influence of various scSE modules or their combinations on the performance of crack detection in the downsampling and upsampling stages. The details are presented in Section 5.

### 3.3. Loss Function

The loss function is a core component of deep learning methods that was used for measuring the deviation between the predicted values and the true values of models and usually served as an objective function of the model optimization. The essence of crack segmentation is to classify each pixel of the pavement image containing cracks as cracks or background. It is worth noting that compared with the pavement background, the cracked pixels only accounted for a small proportion of the whole pavement image. To solve this serious class imbalance problem, we chose focal loss [67] as the loss function. Focal loss was modified based on standard cross-entropy loss. It introduced two penalty factors to reduce the weight of easy-to-classify samples, which made the model focus more on difficult-to-classify samples in the training process. The focal loss could be expressed as

(1) $$FL\left(p,\hat{p}\right)=-\left(\alpha {\left(1-\hat{p}\right)}^{\gamma }plog\left(\hat{p}\right)+\left(1-\alpha \right){\hat{p}}^{\gamma }\left(1-p\right)\log\left(1-\hat{p}\right)\right)$$

where *α* and (1 − *α*) were used to control the proportions of positive and negative samples, respectively, with values ranging from [0, 1]. The parameter *γ* is called the focusing parameter, and its value range was [0, +∞). When *γ* = 0, focal loss degenerated into cross-entropy loss, and the larger *γ* was, the greater the punishment for the easy-to-classify samples would be.

### 3.4. Parameter Optimization

In order to minimize the loss, the Adam optimizer was chosen to iteratively update the model parameters. The Adam optimizer is essentially RMSprop with momentum, which dynamically adjusted the learning rate of each parameter by using the first moment estimation and the second moment estimation of gradient. Its advantage was that after bias correction, the learning rate of each iteration had a certain range, which made the parameters stable. The update process could be simply represented as follows:

(2) $$m_{t}=\beta_{1}m_{t-1}+\left(1-\beta_{1}\right)\nabla_{\theta }J\left(\theta \right)\;v_{t}=\beta_{2}v_{t-1}+\left(1-\beta_{2}\right){\left(\nabla_{\theta }J\left(\theta \right)\right)}^{2}\;{\hat{m}}_{t}=\frac{m_{t}}{1-\beta_{1}{}^{t}}\;{\hat{v}}_{t}=\frac{v_{t}}{1-\beta_{2}{}^{t}}\;\theta_{t}=\theta_{t-1}-\frac{\alpha }{\sqrt{\hat{v_{t}}+\varepsilon }}\hat{m_{t}}$$

where $\;\beta_{1}$ and $\beta_{2}$ represent the exponential decay rates of first-order moment estimation and second-order moment estimation, which are set to 0.9 and 0.99, respectively; *t* is the index of iterations; α represents the learning rate; $m_{t}$ and $v_{t}$ represent exponential moving averages of the first-order and second-order moments of the gradient, respectively; and ${\hat{m}}_{t}$ and ${\hat{v}}_{t}$ are the unbiased values of $m_{t}$ and $v_{t}$, respectively. θ represents the network model parameters that need to be updated by learning [59].

## 4. Experiment Result and Discussion

### 4.1. Implementation Details

The workstation specifications for training our neural network were RTX3090 GPU, Intel i9 processor, and 32GB RAM. The deep learning framework we used was Pytorch version 1.9.0, which is completely open source

The settings of hyperparameters included the following: the basic learning rate was set to 0.0005, the weight decay was set to 0.0001, the batch size was set to 4, and the ‘poly’ learning rate reduction strategy was adopted with power 2.

### 4.2. Datasets

We evaluated our methods using three public datasets: CFD, Crack500, and DeepCrack. The following is a brief description of them.

The CFD dataset, published in [23], consists of 118 RGB images with a resolution of 480 × 320 pixels. All of the images were taken using an iPhone5 smartphone on the road in Beijing, China, and can roughly reflect the existing urban road conditions in Beijing. These crack images have uneven illumination and contain noise such as shadows, oil spots, and lane lines, and most cracks in these images are thin cracks, which make crack detection difficult. We randomly divided 70% of the dataset (82 images) for training and 30% of the dataset (36 images) for testing.

The Crack500 dataset, shared by Yang et al. in the literature [60], contains 500 original images with a resolution of 2560 × 1440 collected at the main campus of Temple University. Each original image was cropped into a non-overlapping image area of 640 × 360, resulting in 1896 training images, 348 validation images, and 1123 test images. These images are characterized by low contrast between cracks and background, as well as noise such as oil pollution and occlusions, which increase the difficulty of detection.

The DeepCrack dataset [2] contains 537 crack images, including both concrete pavement and asphalt pavement, with complex background and various crack widths, ranging from 1 pixel to 180 pixels. We kept the same data split as the original paper, with 300 images for training and 237 images for testing.

We randomly applied data augmentations to each image during training; the main methods included random vertical or horizontal flipping, random brightness and contrast changes, random scaling, and rotation.

### 4.3. Evaluation Criteria

To evaluate the performance of crack detection in this study, we introduced four basic evaluation metrics, precision (Pr), recall (Re), F1 score (F1), and intersection over union (IoU). In the crack segmentation task, crack pixels were defined as positive samples, and non-crack pixels were defined as negative samples. According to ground truth and prediction results, pixels could be divided into four cases, as shown in Table 1.

Then, Pr, Re, F1, and IoU could be defined as

(3) $$P_{r}=\frac{TP}{TP+FP}$$

(4) $$R_{e}=\frac{TP}{TP+FN}$$

(5) $$F1=\frac{2\times P_{r}\times R_{e}}{P_{r}+R_{e}}$$

(6) $$IoU=\frac{GroundTruth\cap Prediction}{GroundTruth\cup Prediction}$$

### 4.4. Experiment Results and Discussion

To verify the crack segmentation effect of the model described in Section 3, we compared it with three classical segmentation algorithms, FCN, SegNet, and U-Net, using the DeepCrack dataset, Crack500 dataset, and CFD dataset, respectively. The following is a comparative analysis and discussion of the experimental results for the three datasets.

#### 4.4.1. Results Using the CFD Dataset

First, we performed experimental verification and comparison using the published CFD dataset, which contained both asphalt cracks and concrete cracks and had an image size of 480 × 320 pixels.

**Figure 3:**
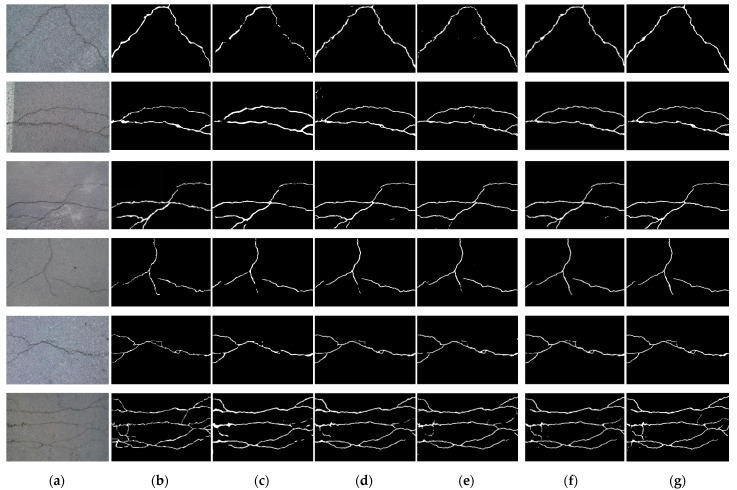
Comparison of predicted results among various methods for the CFD dataset. Columns from left to right: (**a**) original image, (**b**) ground truth, (**c**) FCN, (**d**) Unet, (**e**) SegNet, (**f**) TransUnet, and (**g**) our method.

Figure 3 shows the crack detection results of six typical input images of our method and the three methods to be compared. The first column is the original input crack image, the second column is the label image corresponding to the first column image, and the next four columns are the predicted output images of the four comparison algorithms. As can be seen from Figure 3, all these algorithms could detect the rough crack profile. However, in terms of details, all three algorithms, FCN, Unet, and SegNet, had false detection and missing cracks resulting in discontinuity of cracks to a varying degree. Our algorithm was obviously better than the three algorithms, with the least false detection and missing cracks, and the closest to the ground truth.

As shown in Table 2, we also performed a quantitative comparison of these crack detection algorithms. Our crack segmentation algorithm outperformed all the other algorithms in four metrics: Pr, Re, F1, and IoU.

#### 4.4.2. Results Using the Crack500 Dataset

**Figure 4:**
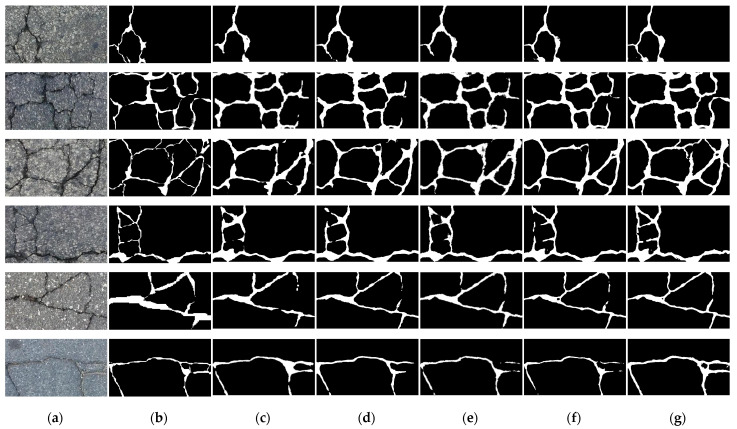
Comparison of predicted results among various methods for the Crack500 dataset. Columns from left to right: (**a**) original image, (**b**) ground truth, (**c**) FCN, (**d**) Unet, (**e**) SegNet, (**f**) TransUnet, and (**g**) our method.

To further compare the detection performance of these algorithms, we conducted experimental verification of the public Crack500 dataset. The images of this dataset were all asphalt cracks, which were complicated in texture, low in contrast, inconspicuous in characteristics, and difficult to detect. The experimental results presented in Figure 4 show that even in this complex case, our algorithm had better robustness and better detection results in comparison.

The quantitative experimental results can be seen in Table 3, where RUC-Net achieved the best performance in all metrics.

#### 4.4.3. Results for the DeepCrack Dataset

**Figure 5:**
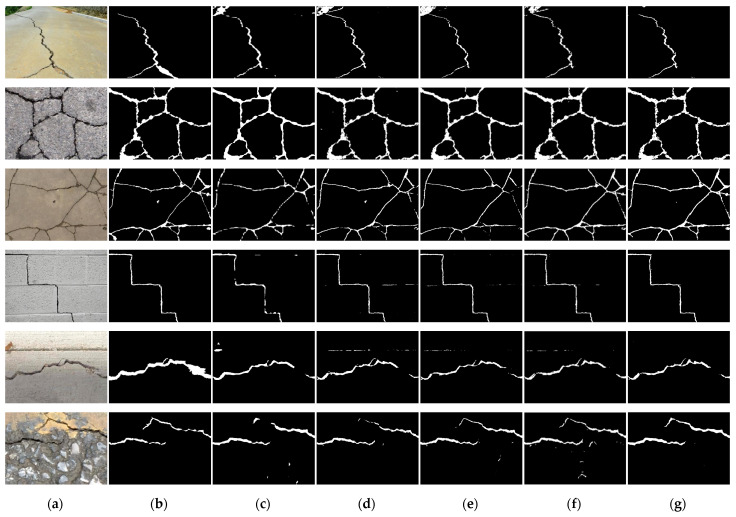
Comparison of predicted results among various methods for the DeepCrack dataset. Columns from left to right: (**a**) original image, (**b**) ground truth, (**c**) FCN, (**d**) Unet, (**e**) SegNet, (**f**) TransUnet, and (**g**) our method.

In this set of comparative experiments, we choose the public dataset DeepCrack for experimental verification. The crack image of the dataset includes asphalt cracks and concrete cracks, and the image size is 544 × 384. As can be seen from the experimental results in Figure 5, our algorithm achieves the relatively best detection performance even in the presence of complex backgrounds and strong interference.

As shown in Table 4, RUC-Net achieves the highest Pr, Re, F1 and IoU compared with other crack segmentation algorithms. The Pr, Re, F1 and IoU reached 88.33%, 81.2% 84.61% and 73.33% respectively.

## 5. Ablation Studies

We conducted ablation studies using the CFD dataset to show the performance improvement of our algorithm design choices.

### 5.1. Effect of Various scSE Modules and Their Combinations on Improving Detection Performance

The scSE module had three variants, sSE, cSE, and scSE. There were many situations using various combinations of scSE modules on the encoder (that is, the downsampling stage) and decoder (that is, the upsampling stage) of RUC-Net. We compared the impacts of these different situations on the pavement crack detection performance. Table 5 shows several typical combinations. As can be seen from the table, except for downcSE, integrating various other scSE modules in RUC-Net could all slightly improve the detection performance. In terms of single cSE or sSE, the upcSE obtained the best results, and in terms of combined strategies, the upscSE achieved the best performance.

### 5.2. Comparison of Various Parameters of the Focal Loss Function

We applied the focal loss function to deal with the class imbalance problem in crack segmentation; the key was to choose the appropriate parameter combination of *α* and *γ*. We chose different parameter combinations of *γ* and *α* for comparative experimental research using the CFD dataset.

The experimental results are shown in Table 6. In most cases, with the increase in α, recall was higher and precision was lower. Under different conditions of *γ* being 1.5, 2, and 2.5, *α* being 0.6 achieved the best results. As far as the average value of F1 scores under different *α* values was concerned, *γ* being 1.5 was superior to *γ* being 2 or 2.5. Obviously, the best parameter combination was *γ* being 1.5 and α being 0.6. This was exactly the parameter combination used in the previous experiments in this paper.

## 6. Conclusions

In this paper, RUC-Net was proposed for pixel-level pavement crack segmentation. The architecture of RUC-Net was a U-shaped encoder–decoder network combining Unet and Resnet. The residual block in ResNet was used to replace the two 3 × 3 convolution layers in the encoder of original Unet, so as to extract more precise crack feature information. In the decoder network part, RUC-Net combined local information in shallow layers and semantic information in deep layers through concatenating to obtain more refined segmentation effects. In addition, we introduced the scSE attention module to enhance important information features while suppressing unimportant information features in space and channels, so as to further improve the crack segmentation effect. The focal loss function was used to deal with the class imbalance problem in crack segmentation. Our approach achieved an F1 score of 73.92% for the CFD dataset, 72.9% for the Crack500 dataset, and 84.61% for the DeepCrack dataset, outperforming FCN, Unet, and SegNet.

One limitation of this research was that our algorithm still needed to manually mark every pixel of the ground truth image, which made data acquisition expensive. To mitigate this issue, it was a research direction to adopt unsupervised learning-based techniques. As the supervised learning algorithm aimed to fit the function that approximated the given labeled training data, the actual performance of this kind of algorithm largely depended on the size and quality of the training dataset. So, establishing a wider, larger, and high-quality dataset and fully investigating data augmentation techniques are also directions we need to work on.

## Figures and Tables

**Table 1:** All the results of the predicted case and the ground truth case.

	Predicted	Crack	No Crack
Ground Truth			
Crack		True positive (TP)	False negative (FN)
No crack		False positive (FP)	True negative (TN)

**Table 2:** The Pr, Re, F1, and IoU of the compared methods for the CFD dataset.

Methods	Pr	Re	F1	IoU
FCN	0.6659	0.7483	0.7047	0.5441
SegNet	0.6799	0.7492	0.7129	0.5539
Unet	0.7008	0.7496	0.7244	0.5679
TransUnet	0.7058	0.7559	0.7300	0.5748
Ours	**0.7125**	**0.7680**	**0.7392**	**0.5863**

**Table 3:** The Pr, Re, F1, and IoU of compared methods for the Crack500 dataset.

Methods	Pr	Re	F1	IoU
FCN	0.6830	0.7206	0.7013	0.5400
SegNet	0.6893	0.7303	0.7092	0.5494
Unet	0.6852	0.7541	0.7180	0.5600
TransUnet	**0.7025**	0.7424	0.7219	0.5648
Ours	0.6988	**0.7619**	**0.7290**	**0.5736**

**Table 4:** The Pr, Re, F1, and IoU of compared methods for the DeepCrack dataset.

Methods	Pr	Re	F1	IoU
FCN	0.8600	0.7737	0.8146	0.6871
SegNet	0.8632	0.7954	0.8279	0.7064
Unet	0.8810	0.7829	0.8291	0.7080
TransUnet	0.8730	0.7976	0.8336	0.7147
Ours	**0.8833**	**0.8120**	**0.8461**	**0.7333**

**Table 5:** The differences of various scSE modules and their combinations in improving the performance of crack detection taking CFD as an example.

Methods	Pr	Re	F1	IoU
RUC-Net	0.7136	0.7633	0.7375	0.5842
RUC-Net+downcSE *	0.7055	0.7596	0.7315	0.5767
RUC-Net+downsSE	0.7092	0.7699	0.7383	0.5851
RUC-Net+upsSE	0.7135	0.7643	0.7381	0.5849
RUC-Net+upcSE	0.7122	0.7676	0.7388	0.5858
RUC-Net+downscSE	0.7099	0.7691	0.7383	0.5852
RUC-Net+upscSE	0.7160	0.7657	0.7398	0.5871
RUC-Net+fullscSE	0.7064	0.7758	0.7395	0.5866

* The downcSE represents using only the sCE module in the downsampling stage, the upsSE represents using only the sSE module in the upsampling stage, and so on, while the fullscSE represents using scSE module both in the upsampling stage and downsampling stage.

**Table 6:** The comparison of parameter combinations of focal loss.

Parameter Combination		Pr	Re	F1	IoU
*γ*	*α*				
1.5	0.5	0.7353	0.7347	0.7349	0.5809
	0.6	0.7160	0.7657	**0.7398**	0.5871
	0.7	0.7017	0.7747	0.7359	0.5822
	0.8	0.6704	0.8058	0.7318	0.5770
2	0.5	0.7347	0.7289	0.7316	0.5768
	0.6	0.7027	0.7776	0.7381	0.5850
	0.7	0.6840	0.7987	0.7369	0.5834
	0.8	0.6697	0.7999	0.7284	0.5729
2.5	0.5	0.7337	0.7293	0.7315	0.5767
	0.6	0.7062	0.7748	0.7389	0.5859
	0.7	0.6867	0.7924	0.7369	0.5834
	0.8	0.6805	0.7825	0.7279	0.5722

## Data Availability

Please contact Gui Yu (yugui@hgnu.edu.cn).
